# Grafting and stabilization of ordered mesoporous silica COK-12 with graphene oxide for enhanced removal of methylene blue†

**DOI:** 10.1039/c9ra05541j

**Published:** 2019-11-07

**Authors:** Laura M. Henning, Ulla Simon, Aleksander Gurlo, Glen J. Smales, Maged F. Bekheet

**Affiliations:** Fachgebiet Keramische Werkstoffe/Chair of Advanced Ceramic Materials, Institut für Werkstoffwissenschaften und-technologien, Fakultät III, Technische Universität Berlin Hardenbergstr. 40 10623 Berlin Germany laura.m.henning@ceramics.tu-berlin.de +49 30 314 25202; Bundesanstalt für Materialforschung und -prüfung (BAM), Division 6.5 – Polymers in Life Sciences and Nanotechnology Unter den Eichen 87 12205 Berlin Germany

## Abstract

Large-pore ordered mesoporous silica (OMS) COK-12, analogous to the well-known SBA-15, but synthesized in a more environmentally friendly way and exhibiting a shorter plate-like structure, was grafted with different amounts of graphene oxide (GO) for the first time in an inexpensive and rapid process, that was successfully upscaled. Samples were examined with nitrogen sorption analysis, SAXS, Raman spectroscopy, XPS, and zeta potential analysis. Adsorption experiments with the cationic dye methylene blue (MB) were conducted on the grafted materials and on pure COK-12, taking into account the influence of initial dye concentration (30–600 mg L^−1^), adsorbent dosage (0.2–14 g L^−1^), contact time (0.3–300 min), solution pH (4–10), and influence of salts and temperature (0–1 M NaCl, 80 °C) to simulate industrial dye effluent. The adsorption process was found to be represented best by the Langmuir isotherm model, *i.e.*, adsorption is a monolayer process. The calculated maximum adsorption capacities were found to be 20.2 and 197.5 mg g^−1^ at dosages of 5 and 0.5 g L^−1^ for pure COK-12 and COK-12 grafted with 50 wt% GO, respectively, at pH 5.65 and MB concentration of 100 mg L^−1^. Adsorption kinetics were found to follow the pseudo-second order model, *i.e.*, chemisorption is the rate controlling step. The adsorption performances could be well preserved at simulated dye effluent. Desorption was found to be most effective with hydrochloric acid. The COK-12 grafted with GO presented in this work shows superior adsorption properties in comparison to other grafted OMS materials. In addition, grafting with GO remarkably improved the stability of COK-12 in aqueous solution.

## Abstract 2

Grafting of environmentally friendly produced ordered mesoporous silica (OMS) COK-12 with graphene oxide yields a superior OMS/GO adsorbent for methylene blue.

## Graphical abstract

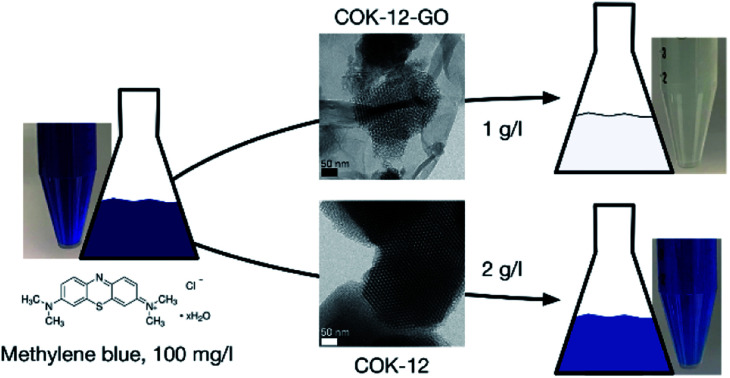

## Introduction

The textile industry is one of the main generators of liquid effluent pollutants. The total worldwide consumption of dyes by the textile industry exceeds ten thousand tons per year, from which approximately one thousand tons are discharged into rivers and streams. Most of the dyes used by the textile industry and other industries are toxic and can cause severe problems to both humans and the aquatic environment. Whilst humans can develop nausea, skin irritations, hemorrhages, and/or cancer, the partial blocking of sunlight due to the dark color of the effluents can hinder the life of aquatic organisms and reduce photosynthesis.

Different approaches have been utilized for the removal of dyes from aqueous solutions. Chemical treatments such as oxidative processes, especially heterogeneous photocatalytic oxidation, or the usage of Fenton's reagent, whilst simple, can degrade the dyes, but usually consume a lot of energy or generate byproducts and sludge that cannot be reused.^1^ Hence, recent research focuses on highly reactive photocatalytic systems that maximize the use of illumination wavelengths in the visible light region^2^ or novel catalytic core–shell nanocomposites that can successfully degrade dyes.^3^ Biological methods mainly use fungi or bacteria for the degradation of dyes, making them ecologically more favorable.^4^ However, they are not effective for all dyes and the breakdown of some dyes by biological methods can yield harmful substances like methane or hydrogen sulfide.^5^ Physical treatments, such as membrane filtration has the advantage of continuously separating the dye from the effluent, but has high capital and maintenance costs, as clogging can occur.^6^ On the other hand, adsorption is recognized as the most promising method for dye removal because of its versatility, wide applicability, and low cost as most adsorbents are easily available.^7^ However, practical application has been limited by regeneration problems, disposal, poor mechanical stability, or low removal effectiveness for a wide range of dyes. In the past few decades several attempts were made to overcome these problems by using nanomaterials exhibiting high surface areas.^8^

Due to their extremely high surface area, tunable pore size, and high adsorption capacity ordered mesoporous silica (OMS) materials have gained interest in the field of dye adsorption. Pure OMS materials like MCM and SBA have been used for the adsorption of dyes, but also modified OMS materials with grafted functional groups like carboxylic and amino groups or transition metal oxides have been utilized to enhance adsorption.^9^ Furthermore, several carbon-based materials such as activated carbon, carbon nanotubes, and graphene oxide (GO) have been reported for the adsorption of many organic dyes as well as heavy metals.^10,11^ Among these materials has GO, an oxidized form of graphene, received considerable attention in the field of adsorption due to its high specific surface area as well as its numerous functional groups such as hydroxyl, carboxyl, and epoxyl groups. The ionization of these functional groups on the GO surface in aqueous solutions makes its surface highly negative, which in turn facilitates its interactions with positively charged organic molecules and metal cations. However, the extraction of pure GO from aqueous solution after the adsorption process is very difficult due to its strong hydrophilicity and stability in water. Thus, GO is usually separated from water by centrifugation using very high forces, *i.e.*, 10 000 rpm, for long durations, thereby consuming a great deal of energy and time.^12^ Hence, composites of GO with other materials such as chitosan hydrogels or polysiloxane gels have been explored as alternative adsorbents for easy separation.^13,14^ Although grafted GO/OMS materials could be equally or more effective and cheap adsorbents, only few examples exist in literature dealing with grafting of GO on OMS materials.^15–18^ COK-12, an additional type of OMS material, exhibiting short plate-like particles, has been chosen for this work as it can be produced from the same nonionic triblock copolymer as SBA-15, at room temperature under quasi neutral pH using an inexpensive silica precursor, making the synthesis more environmentally friendly in comparison to other OMS materials.^19^ Although COK-12 typically has a lower specific surface area than SBA-15, recent improvements to the specific surface area, pore size, and pore volume through controlling synthesis steps and parameters means that the properties of COK-12 are now competitive with those of SBA-15.^20,21^ Furthermore, successful upscaling of the COK-12 synthesis has also been demonstrated.^22,23^

In this work an inexpensive and rapid surface GO grafting process was applied to more environmentally friendly synthesized mesoporous silica COK-12 and successfully upscaled for the first time. This process allows to obtain a superior dye adsorbent while overcoming the drawbacks of energy intensive separation of pure GO from aqueous solutions and the insufficient stability of amorphous silica.

## Experimental

### Chemicals

The templating agent Pluronic P123 (MW ∼ 5800 g mol^−1^), sulfuric acid (95–98%), and methylene blue (MB) (98%) were obtained from Sigma-Aldrich (Merck, Germany). Citric acid (≥99.5%, anhydrous), trisodium citrate dihydrate (≥99%), hydrochloric acid (37%), hydrogen peroxide (30%), potassium permanganate (≥99%), and sodium silicate (7.8–8.5 wt% Na_2_O, 25.8–28.5 wt% SiO_2_) were purchased from Carl Roth GmbH + Co. KG (Germany). Graphite (KS6) and phosphoric acid (85%) were acquired from Lonza (Switzerland) and Chemsolute (Th. Geyer GmbH + Co. KG, Germany), respectively. Sodium hydrogen carbonate (ACS Reag. Ph Eur), sodium hydroxide, acetic acid (100%, p.a.), 1-butanol (100%), and toluene (≥99%) were obtained from Merck. Deionized water (DIW) was used for all syntheses.

### COK-12 synthesis

The synthesis of COK-12 was performed based on the procedure introduced by Jammaer *et al.*^19^ with an additional aging step and thorough washing to achieve a higher specific surface area and pore volume as recently published.^20^ In a typical synthesis, 4 g of P123 was dissolved in 107.5 mL DIW under continuous stirring. After complete dissolution of P123, 3.362 g anhydrous citric acid and 2.882 g trisodium citrate dihydrate were added to buffer the pH of the synthesis (pH ∼ 5). The buffered surfactant solution was then stirred for 24 h. A solution of 10.4 g sodium silicate and 30 mL DIW was prepared and incorporated into the buffered micellar solution. Immediate solid formation was observed and stirring was maintained for 5 min after which the slurry was aged for 24 h without stirring. Afterwards, the additional aging step was conducted, where the slurry was kept at 90 °C for further 24 h within a closed system to avoid solvent loss due to evaporation. Subsequently, the solid was separated from the synthesis solution by vacuum filtration and gradually washed with 500 mL DIW before being dried at 60 °C overnight. The dry solid was then calcined in air at 500 °C with a 1 K min^−1^ heating ramp and 8 h dwell time to completely remove the organic templating agent.

### Graphene oxide (GO) synthesis

The synthesis of graphene oxide (GO) was performed based on the modified Hummers' method as reported by Marcano *et al.*^24^ Therefore, a mixture consisting of 360 mL sulfuric acid and 40 mL phosphoric acid was slowly poured on 3 g graphite inside a round bottom flask in a reflux system under stirring. Afterwards, 18 g of potassium permanganate were added to the solution very slowly. The temperature of the mixture was then raised to 50 °C and held for 12 h in total. Subsequently, the solution was stirred on 1 l of ice prepared from DIW while 8 mL of hydrogen peroxide were added dropwise. Finally, the GO was washed with hydrochloric acid (15%) and DIW until the pH was quasi neutral and then freeze-dried in a VaCo 5 at −92 °C and 0.158 mbar for 72 h (Zirbus Technology, Germany).

### Grafting of COK-12 with GO

COK-12 was modified with GO by direct grafting according to the procedure published by Mirabi *et al.* for SBA-15.^25^ Suspensions of 0.1 g of COK-12 and 25 mL 0.16 M sodium hydrogen carbonate each were prepared and mixed thoroughly using a vortex mixer and a tube mixer for 25 min. Afterwards, the suspensions were centrifuged for 15 min at 3600 rpm in a Jouan C414. The supernatants were decanted and the materials washed with DIW twice. Meanwhile, GO was suspended in 25 mL toluene. Therefore, different amounts of GO were used, namely 0.1 g, *i.e.*, 50 wt%, as used by Mirabi *et al.*, as well as lower and higher mass fractions of 0.004 g, *i.e.*, ∼4 wt%, and 0.15 g, *i.e.*, 60 wt%. The GO suspensions were treated with ultrasound for 3 h in 30 min intervals, poured onto the wet COK-12 and mixed with a vortex mixer. Immediate solid formation took place. For grafting the highest concentration of GO, *i.e.*, 60 wt%, it could be observed that some GO deposited on the inner wall of the centrifuge tube and did not attach to the COK-12. Finally, the mixtures were centrifuged as described above, the supernatant was decanted, and the powders were dried in air. The resulting materials were named COK-12-GO-1, -2, and -3 for the 4, 50, and 60 wt% GO concentration, respectively. In addition, for the medium GO concentration, *i.e.*, 50 wt%, an upscaling of the grafting process by a factor of five was realized, which is labelled with an asterisk (*) in the sample name.

### Dye adsorption experiments

#### Batch equilibrium studies

An aqueous stock solution of the cationic dye MB with a concentration of 1000 mg L^−1^ was prepared by dissolving 1 g of the dye in 1 L DIW. Solutions with lower dye concentration were prepared from this stock solution. Adsorption experiments were performed in 50 mL beakers with 10 mL dye solution at 25 °C and 450 rpm. In first batch adsorption experiments, the pure COK-12 and the GO grafted COK-12 materials were tested with a dosage of 2 g L^−1^ with an initial dye concentration of 100 mg L^−1^ at pH value 5.65 for 180 min. Further experiments, performed to study the influence of dosage, pH, time, initial dye concentration, and influence of salts on the adsorption process, were conducted on the pure COK-12 and the upscaled grafted COK-12-GO-2*. The effect of adsorbent dosage was studied in the range of 0.5 to 14 g L^−1^ and 0.2 to 3 g L^−1^ for COK-12 and COK-12-GO-2*, respectively. The pH was adjusted between 4.1 and 9.8 using HCl and NaOH. Following the adsorption experiments, the adsorbent was separated from the dye solution by centrifugation at 3600 rpm in a Jouan C414. ICP-OES measurements were performed to determine the Si content. The dye concentration in the supernatant was determined by UV-Vis spectroscopy at 664 nm. The removal efficiency *R*_e_ in % and the equilibrium adsorption capacity *q*_e_ in mg g^−1^ were calculated as given in eqn (1) and (2), respectively,1
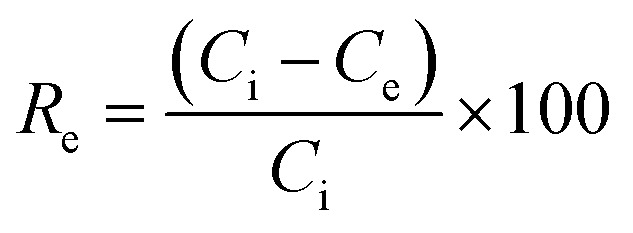
2
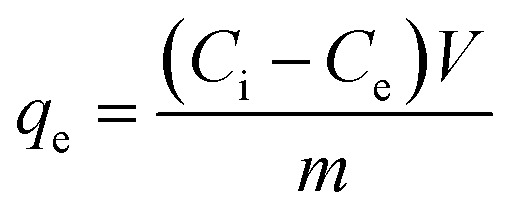
whereby *C*_i_ and *C*_e_ are the initial and equilibrium mass concentration of the dye in the solution in mg L^−1^, *m* is the mass of the adsorbent in g, and *V* is the volume of the dye solution in L.

### Adsorption isotherms

Dye concentrations between 30 and 600 mg L^−1^ were used to obtain adsorption isotherms. The dosage was 5 and 0.5 g L^−1^ for COK-12 and COK-12-GO-2*, respectively. 10 mL of the respective dye solution and a magnetic stir bar were put in a 50 mL beaker at 25 °C. The stirring speed was set to 450 rpm. To the dye solution, the respective amount of adsorbent was added. The beaker was covered and stirring was maintained for 180 min. The experimental adsorption data was fitted using the non-linear form of the Langmuir and Freundlich isotherm models expressed by eqn (3) and (4), respectively,3
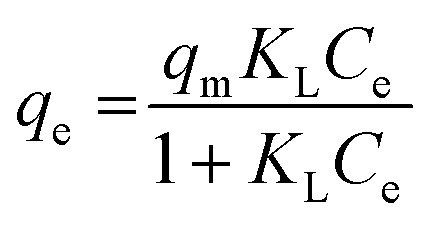

4 *q*_e_ = *K*_F_*C*_e_^1/*n*^

whereby *q*_e_ is the equilibrium adsorption capacity in mg g^−1^, *C*_e_ is the equilibrium dye concentration in mg L^−1^, *q*_m_ is the maximum adsorption capacity in mg g^−1^, *K*_L_ is the Langmuir isotherm constant in L mg^−1^, and *K*_F_ and *n* are the Freundlich coefficients in mg g^−1^ (L mg^−1^)^1/*n*^ and mg g^−1^, respectively.

### Adsorption kinetics

To study the adsorption kinetics, contact times between 0.3 and 300 min were tested. The dosage was 5 and 0.5 g L^−1^ for COK-12 and COK-12-GO-2*, respectively, and the initial dye concentration was 100 mg L^−1^. 10 mL of the dye solution and a magnetic stir bar were placed in a 50 mL beaker at 25 °C. The stirring speed was set to 450 rpm. To the dye solution, the respective amount of adsorbent was added. The beaker was covered and stirring was maintained for the respective times. To analyze the adsorption kinetics, the pseudo-first-order model (5), pseudo-second-order model (6), and Elovich model (7) were applied,

5 *q*_*t*_ = *q*_e_(1 − e^−*K*_1_*t*^),

6
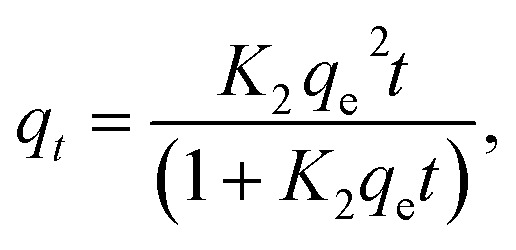
7
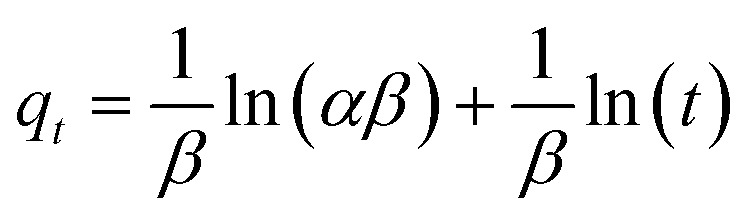
whereas *t* is the time in min, *K*_1_ and *K*_2_ are the pseudo-first order and pseudo-second order constants in min^−1^ and g mg^−1^ min^−1^, respectively, and *α* and *β* are the initial adsorption rate and the Elovich desorption constant, respectively.

### Adsorption behavior under simulated textile effluent conditions

To simulate textile effluent conditions, adsorption experiments were conducted at 80 °C and in the presence of salts (0–1 M NaCl). The dosage was 5 and 0.5 g L^−1^ for COK-12 and COK-12-GO-2*, respectively. The MB concentration was 100 mg L^−1^ and the pH 5.65. Stirring was maintained for 180 min at 450 rpm.

### Dye recovery from the adsorbents

Batch mode adsorption was performed as described above with dosages of 5 and 0.5 g L^−1^ for COK-12 and COK-12-GO-2*, respectively. The adsorbents were then dried and 10 mL of the desorption agents DIW, 1-butanol, acetic acid, 0.1 M HCl, and concentrated HCl, respectively, were added. Stirring was maintained for 180 min at 25 °C and 450 rpm.

### Characterization

The pore structure, pore size, and specific surface area was studied using nitrogen sorption analysis in a QuadraSorb Station 4 apparatus (Quantachrome, USA). Isotherms were recorded at 77 K after degassing under vacuum for 10 h at 200 °C and room temperature for COK-12 and COK-12-GO samples, respectively. The surface area was determined using the Brunauer, Emmett and Teller (BET) method. The estimation of the size of the mesopores was based on non-local density function theory (NLDFT) calculations using the adsorption branch of the isotherm. Micropores were analyzed by the t-plot method. All nitrogen sorption data was analyzed using the Quantachrome/QuadraWin software version 5.05.

Long-range ordering was studied by small angle X-ray scattering (SAXS) in Multiscale Analyser for Ultrafine Structures (MAUS): a heavily customized Xeuss 2.0 (Xenocs, France). X-rays are generated from a microfocus X-ray tube with a copper target, followed by a multilayer optic to parallelize and monochromatize the X-ray beam to a wavelength of 0.154 nm. The detector consists of an in-vacuum motorized Eiger 1 M, placed for this investigation at a distance of 558 mm from the sample. The space between the start of the collimation until the detector is a continuous, uninterrupted vacuum to reduce background. The powders were fixed between two pieces of Scotch Magic tape, in the evacuated sample chamber. The resulting data has been processed and scaled to absolute units using the DAWN software package according to standardized procedures.^26,27^

The wall area, *w*, of the COK-12 was calculated as proposed by Nada *et al.*^28^*via*8
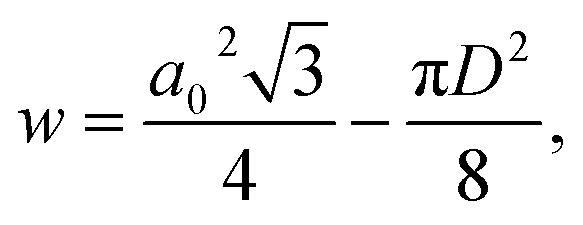
where *a*_0_ is the lattice parameter obtained by SAXS measurements and *D* is the pore diameter obtained from nitrogen sorption measurements, see Fig. S1† for a schematic drawing and derivation of this equation.

The morphology and pore structure of the samples were studied *via* transmission electron microscopy (TEM) in a FEI Tecnai G2 20 S-TWIN equipped with a LaB6-source at 200 keV acceleration voltage (FEI, USA). Images were recorded with a GATAN MS794 P CCD-camera.

The composite structure of COK-12-GO-2* was studied with scanning electron microscopy (SEM) in a Leo Gemini 1530 (Zeiss, Germany) at 5 kV with an in-lens detector. Therefore, the material was immobilized on conducting carbon pads and sputtered with a thin layer of gold.

Inductively coupled plasma optical emission spectroscopy (ICP-OES) at 212.412 nm was performed for elemental analysis of silicon in a Horiba Scientific ICP Ultima2 (Horiba, Japan). The elemental analysis of carbon was performed in a Thermo FlashEA 1112 Elemental Analyzer (Thermo Fisher Scientific, Germany).

Raman spectroscopy has been conducted on a LabRAM 300 (Horiba, Japan) equipped with a He/Ne laser with a wavelength of 632.81 nm. The blaze grid, gap, and spectral center were set to 950 lines mm^−1^, 200 μm, and 1700 cm^−1^, respectively. Five measurements were performed per sample from which LabSpec 5 directly calculated the average. The pure COK-12 and GO grafted samples were measured for 60 s and 20 s, respectively. Pure GO was measured for 3 s. Si(111) was used as a reference material and resulting data was corrected accordingly.

The concentration of MB was determined by UV/vis spectroscopy at 664 nm using a Lambda 900 (PerkinElmer, USA).

Streaming potential measurements were performed on a Stabino system (Particle Metrix, Germany). Samples were measured with a concentration of 3 mg mL^−1^ in 0.1 mM KCl solution. The volume was 10 mL per measurement. Prior to the measurement, the samples were treated in an Sonorex RK 106 ultrasonic bath (Bandelin, Germany) for 5 min. For each sample, two measurements were conducted, one towards acidic conditions until the isoelectric point was reached and the other towards basic conditions up to pH 8.5. Titration was performed in 10 μL intervals using 0.1 M HCl and 0.1 M NaOH solutions. The streaming potential, zeta potential, pH, temperature, and conductivity were recorded 60 s after each titration point to ensure equilibrium.

X-ray photoelectron spectroscopy (XPS) measurements were performed on a K-Alpha (Thermo Fischer Scientific, USA) equipped with a monochromatic Al Kα source. The samples have been prepared on carbon pads. Scans were recorded in the constant analyzer energy mode with a pass energy of 50 eV, step size of 0.1 eV, and with a spot site of 400 μm. All XPS spectra were calibrated using the C1s core line with a binding energy of 284.8 eV. Deconvolution of the spectra was performed by fitting with the Gauss function taking into account a shared full width at half maximum (FWHM).

## Results and discussion

### Adsorbent properties

**Fig. 1:**
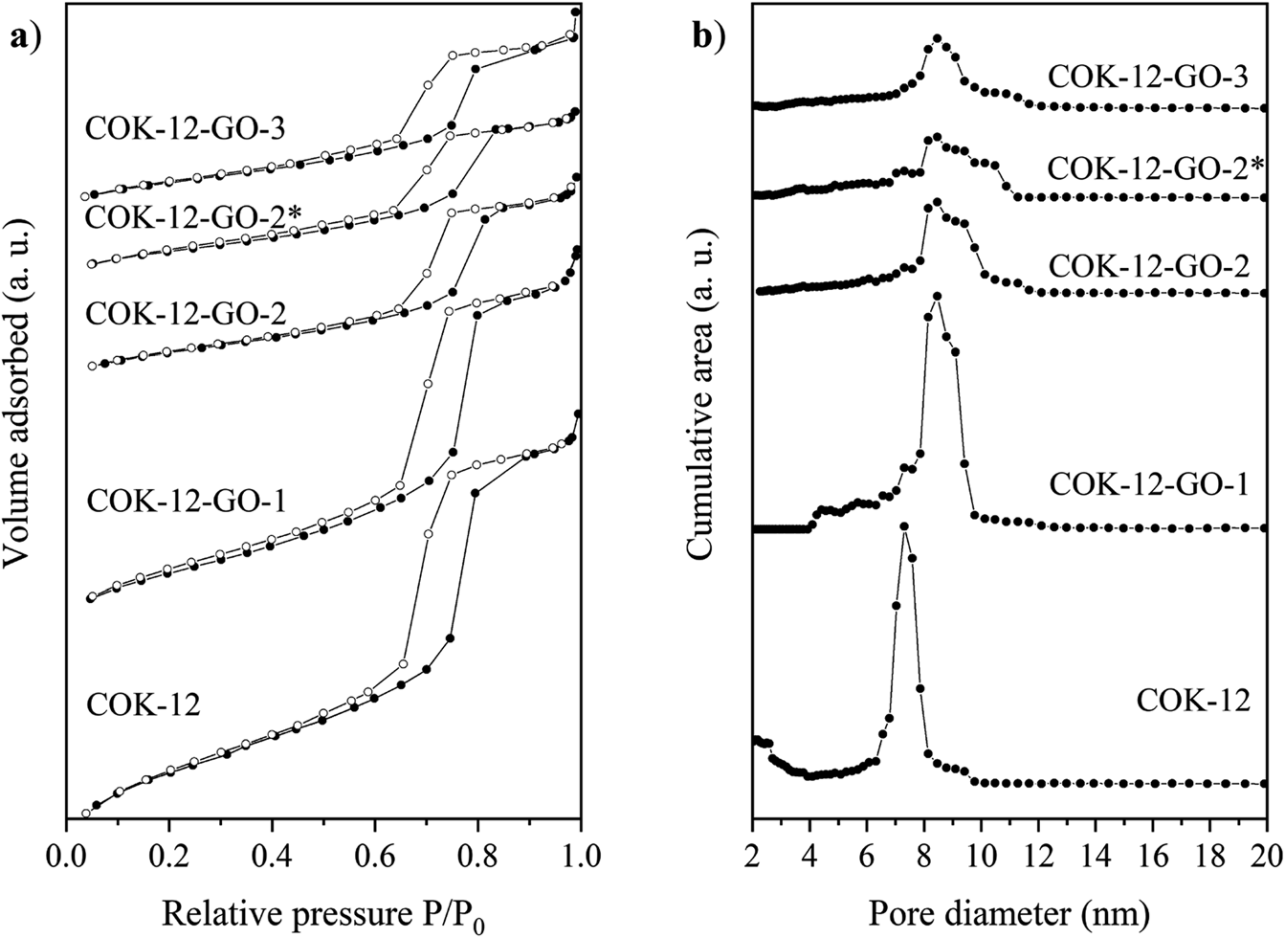
(a) Nitrogen sorption isotherms with filled and open symbols indicating the adsorption and desorption stage, respectively, and (b) pore size distribution of the pure COK-12 and the GO grafted COK-12-GO with low (-1), medium (-2) and high (-3) GO concentration. The asterisk (*) represents the upscaling.

**Fig. 2:**
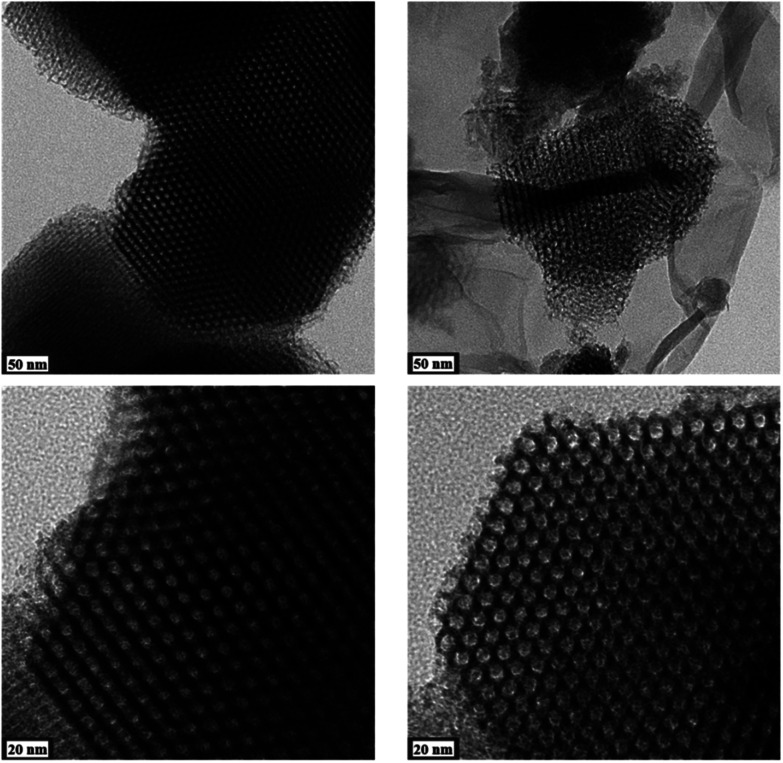
TEM images from the pure COK-12 (left) and the GO grafted COK-12-GO-2 (right).

**Fig. 3:**
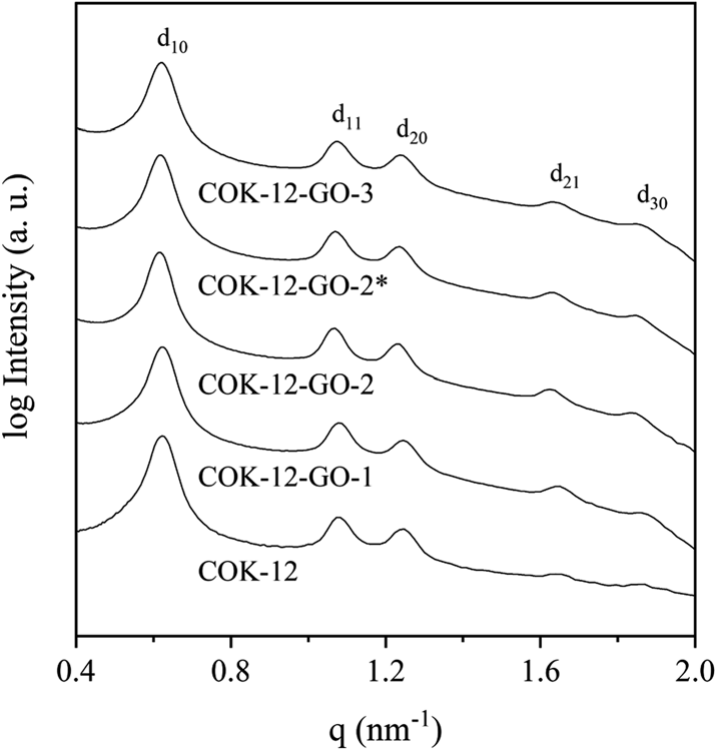
On *d*_10_ normalized SAXS patterns from the pure COK-12 and the GO grafted COK-12-GO with low (-1), medium (-2) and high (-3) GO concentration. The asterisk (*) represents the upscaling.

Results from nitrogen sorption measurements, depicted in Fig. 1, show type IV(a) isotherms with H1 hysteresis loops for both, the pure COK-12 and the GO grafted samples. This is characteristic for mesoporous materials exhibiting uniform pore sizes greater than 4 nm.^29^ Partial blocking of the pores due to the grafting process can be concluded from the decreasing hysteresis areas with increasing GO content. As shown in Table 1, the BET specific surface area decreases with an increase in the amount of grafted GO, namely from 862 m^2^ g^−1^ for the pure COK-12 to 554, 323, and 250 m^2^ g^−1^ for COK-12 with 4, 50, and 60 wt% GO, respectively. Regardless of the GO concentration, the grafted COK-12-GO samples exhibit the same mean pore diameter of 8.5 nm, which is slightly higher than pore diameter of the ungrafted COK-12 with 8.1 nm, indicating that the increase in the pore diameter of the grafted samples is due to the treatment of COK-12 in sodium hydrogen carbonate solution prior to GO grafting. In contrast, the overall accessible pore volume decreases from 1.23 for the pure COK-12 to 1.03, 0.56, and 0.4 cm^3^ g^−1^ for COK-12-GO grafted with GO concentrations of 4, 50, and 60 wt%, respectively. The given results indicate that the decrease in specific surface area of the grafted COK-12-GO samples is due to partial blocking of the pores with GO sheets on the surface and that no GO was incorporated inside the pores. Moreover, the BET specific surface area, pore diameter, and pore volume of the upscaled COK-12-GO-2* were found to be very close to those of COK-12-GO-2 prepared by regular batch size. The slight decrease in BET specific surface area and pore volume with the upscaling can be attributed to the less efficient unfolding of the GO sheets during ultrasonic treatment. The morphology of the GO-grafted COK-12-GO-2* is shown in Fig. S2.† The GO sheets are distributed between COK-12 particles. TEM images of the pure COK-12 and COK-12-GO-2 sample grafted with 50 wt% GO are depicted in Fig. 2. For both samples, the hexagonal pore ordering and overall homogeneous pore size can be observed. In addition, for the grafted COK-12-GO-2 sample thin sheets of GO are present on the surface of COK-12 particles. The hexagonal pore ordering is confirmed by SAXS measurements as depicted in Fig. 3. At least three well resolved reflections can be observed in all SAXS patterns, which can be assigned to the interplanar spacings *d*_10_, *d*_11_, and *d*_20_, as they occur in the ratio of 
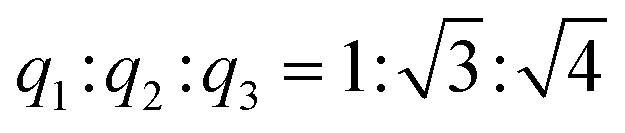
, proving the two dimensional hexagonal *p*6*m* symmetry.^30^ As shown in Table 1, no remarkable changes in the *d*_10_ spacings and lattice parameters can be observed in the course of grafting GO on the surface of COK-12, suggesting that the grafting process did not change the hexagonal pore structure of the material. Moreover, the wall area *w*, see eqn (8), was decreased from 33.5 for COK-12 to 30.9–31.9 nm^2^ for the grafted COK-12-GO samples, compare Table 1. The smaller wall area for the grafted COK-12-GO samples in comparison to the COK-12, which can also be observed in the TEM images in Fig. 2, can be explained by the pore enlargement in the grafted samples due to the treatment with sodium hydrogen carbonate prior to the grafting process. The results confirm that grafting of GO sheets takes place on the surface of COK-12 rather than that GO is incorporated inside the pores.

**Table 1:** Summary of the properties of pure COK-12 and GO grafted COK-12-GO with low (-1), medium (-2) and high (-3) GO concentration. The asterisk (*) represents the upscaling

Sample	*S*_BET_ a (m^2^ g^−1^)	*D* b (nm)	*V*_total_ c (cm^3^ g^−1^)	*q*_10_ d (nm^−1^)	*d*_10_ d (nm)	*a*_0_ e (nm)	*w* f (nm^2^)	*C* g (at%)
COK-12	862	8.1	1.23	0.622	10.1	11.7	33.5	0
COK-12-GO-1	554	8.5	1.03	0.623	10.0	11.7	30.9	4.1
COK-12-GO-2	323	8.5	0.56	0.616	10.2	11.8	31.9	22.5
COK-12-GO-2*	298	8.5	0.48	0.616	10.2	11.8	31.9	21.8
COK-12-GO-3	277	8.5	0.45	0.620	10.1	11.7	30.9	27.4

a Specific surface area estimated by BET method.

b Pore diameter estimated by NLDFT on N_2_-adsorption branch.

c Pore volume estimated by NLDFT.

d Scattering vector *q*_10_ and *d*_10_ spacing determined from SAXS.

e Lattice parameter for the hexagonal symmetry calculated from *d*_10_.

f Wall area calculated according to eqn (8).

g Carbon content from elemental analysis. Carbon content from GO is 44.3 at%.

The Raman spectra of GO, COK-12, and the GO grafted COK-12-GO samples are depicted in Fig. S3.† Characteristic D and G bands at 1350 and 1590 cm^−1^, respectively, are visible for GO as well as for all GO grafted COK-12-GO samples, confirming the presence of GO after the grafting process. In contrast, no D and G bands are detected in the Raman spectrum of pure COK-12, indicating the absence of GO or any other carbon residue from the templating agent P123. The results are in good agreement with those obtained from elemental analysis, which show the absence of carbon for the pure COK-12 and carbon contents of 4–27 at% for the GO grafted COK-12-GO samples, compare Table 1.

**Fig. 4:**
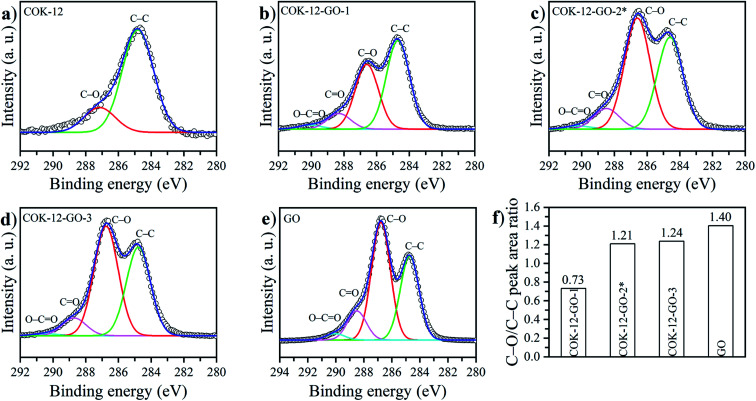
High resolution X-ray photoemission C1s spectra of (a) pure COK-12, and GO grafted COK-12-GO samples with (b) 4 wt%, (c) 50 wt%, and (d) 60 wt% GO as well as (e) pure GO. (f) Illustrates the C–C/C–O peak area ratio for the GO grafted samples and pure GO.

The deconvoluted high-resolution XPS spectra of C1s for GO, ungrafted COK-12, and the grafted COK-12-GO samples, are shown in Fig. 4a–e. It can be seen that all samples show common peaks at 284.8 eV and 286.5 eV, which correspond to C–C and C–O single bond components, respectively.^31^ No other peaks can be observed in the C1s spectrum of the pure COK-12. As revealed by Raman spectroscopy (Fig. S3†) and elemental analysis (Table 1), pure COK-12 does not contain any carbon residue. Thus, these peaks do not reflect the COK-12 properties but rather originate from the carbon pads used in the XPS sample preparation and its partial oxidation. For the pure GO and GO grafted samples, not only peaks originating from the C–C and C–O single bond components of hydroxyl, carboxyl, and 1,2-epoxide functionalities can be observed, but also two additional shoulders at 288.6 eV and 290.0 eV, which are in accordance with the GO spectrum and can be attributed to C=O and O–C=O bonds of carbonyl and carboxyl groups, respectively.^32^ The C–C/C–O peak area ratio indicates the amount of GO grafted on the surface of COK-12 and is depicted in Fig. 4f. With increasing amount of GO used for grafting, *i.e.*, from 4 wt% to 50 wt% GO, the C–C/C–O peak area ratio increases from 0.73 to 1.21, respectively. With further increase of the amount of GO used for grafting, from 50 wt% to 60 wt%, comes a less pronounced increase in the C–C/C–O peak area ratio from 1.21 to 1.24, respectively, indicating that a saturation of COK-12 with GO is almost achieved at a GO concentration of 50 wt%. Overall, it can be concluded that the oxygenated groups from the GO are preserved during the grafting and thus, are available for dye adsorption. Additional XPS Si2p and O1s spectra are depicted in Fig. 11. When comparing the Si2p (oxide) peak of COK-12 before and after GO grafting it can be seen that it is shifted from 103.9 eV towards a lower binding energy of 103.8 eV, indicating a chemical bond between the COK-12 and the GO.

In order to understand the mechanism of dye adsorption on the surface of pure COK-12 as well as of grafted COK-12-GO samples, the charges on their surface were characterized as a function of pH by zeta potential measurements, shown in Fig. S4.† The surface of pure COK-12 possesses its isoelectric point at pH 2.8, which is in accordance with values reported for other OMS materials such as 2.5 for MCM-41 and 2.7 for SBA-15.^33,34^ Above pH 2.8, the surface charge of pure COK-12 becomes negative and reaches a plateau at −58 mV for pH > 6. This result is in good accordance to the behavior of other amorphous silica found in literature.^35^ On the other hand, pure GO exhibits its isoelectric point at pH 2.4 and then shows a rapid decline in the zeta potential up to pH 3.5, followed by a plateau at −80 mV. This plateau for GO is known in literature, but usually drops until −55 mV.^36,37^ The positive charge on the surface of pure COK-12 and GO at pH values lower than the isoelectric point can be explained by the protonation of the hydroxyl, carboxyl, and epoxy groups on the surface in acidic solution. At higher pH values, these functional groups on the surface of COK-12 and GO can deprotonate by interaction with the free hydroxyl ions in basic solution, leading to negatively charged surfaces. Fig. S4† shows that the grafted COK-12-GO-2* sample has an isoelectric point similar to that of pure GO. Moreover, its surface zeta potential shows a similar behavior to that of the pure GO up to pH 3.5. This observation suggests a strong bonding between the COK-12 and the GO as no contribution of the surface charge of the silica can be observed. This finding is in good accordance with the energy shift observed in the XPS Si2p spectra before and after GO grafting (Fig. 11a). However, above pH 3.5, the bonds between the GO and the COK-12 seem to be weakened due to deprotonation of the surface functional groups in both materials. Therefore, the zeta potential of grafted COK-12-GO-2* becomes less negative in comparison to the GO and partially starts to behave more like pure COK-12.

### Adsorption of methylene blue

#### Effect of grafted GO content on COK-12

The effect of the GO concentration used during the grafting of COK-12 on the removal efficiency of MB from water as well as on the stability of the adsorbents during the adsorption experiments using an initial dye concentration of 100 mg L^−1^ at pH 5.65 and contact time of 180 min are depicted in Fig. S5.† At a dosage of 2 g L^−1^, all grafted COK-12-GO samples were able to remove the MB completely, with removal efficiencies between 99.1 and 100%, whereas pure COK-12 could only remove 43% of the MB. Consequently, the grafted COK-12-GO samples show much higher equilibrium adsorption capacities *q*_e_ of 50 mg g^−1^ than the pure COK-12 with 21.5 mg g^−1^. These results can be explained by the higher amount of negative charges on the surface of grafted COK-12-GO samples in comparison with the surface of the pure COK-12, see Fig. S4.† Moreover, pure COK-12 has only hydroxyl groups on the surface, while the surface of the grafted COK-12-GO samples are expected to contain additional carboxyl and epoxy functional groups. Accordingly, the grafted COK-12-GO samples have more negative sites on the surface which can electrostatically interact with the positively charged (=N(CH_3_)_2_)^+^ and (=S–)^+^ groups of the MB. In addition, grafting of COK-12 with 50–60 wt% GO remarkably increases the stability of the silica at the conditions of this adsorption experiment, which was evaluated from the percentage of Si dissolved during the dye adsorption process. While for the pure COK-12 more than 4% of the Si was dissolved and thus, found in the supernatant after centrifugation, whilst less than 0.4 and 0.2% of the Si was dissolved for the samples grafted with 50 and 60 wt% GO, respectively. For the sample grafted with only 4 wt% GO, the stability is decreased, and 6% of the Si was detected in the supernatant. This can be attributed to the treatment with sodium hydrogen carbonate, which deprotonates the hydroxyl groups in order to generate oxygen ions for the grafting process, thus, interfering with the stability of COK-12. With only a very low GO concentration, not all of these locations could be re-stabilized. Thus, for the following experiments, the sample grafted with 50 wt% GO was chosen because of its high removal efficiency as well as its stability in aqueous solution. Furthermore, all of the GO was successfully grafted in contrast to the sample grafted with 60 wt% GO.

#### Effect of adsorbent dosage

**Fig. 5:**
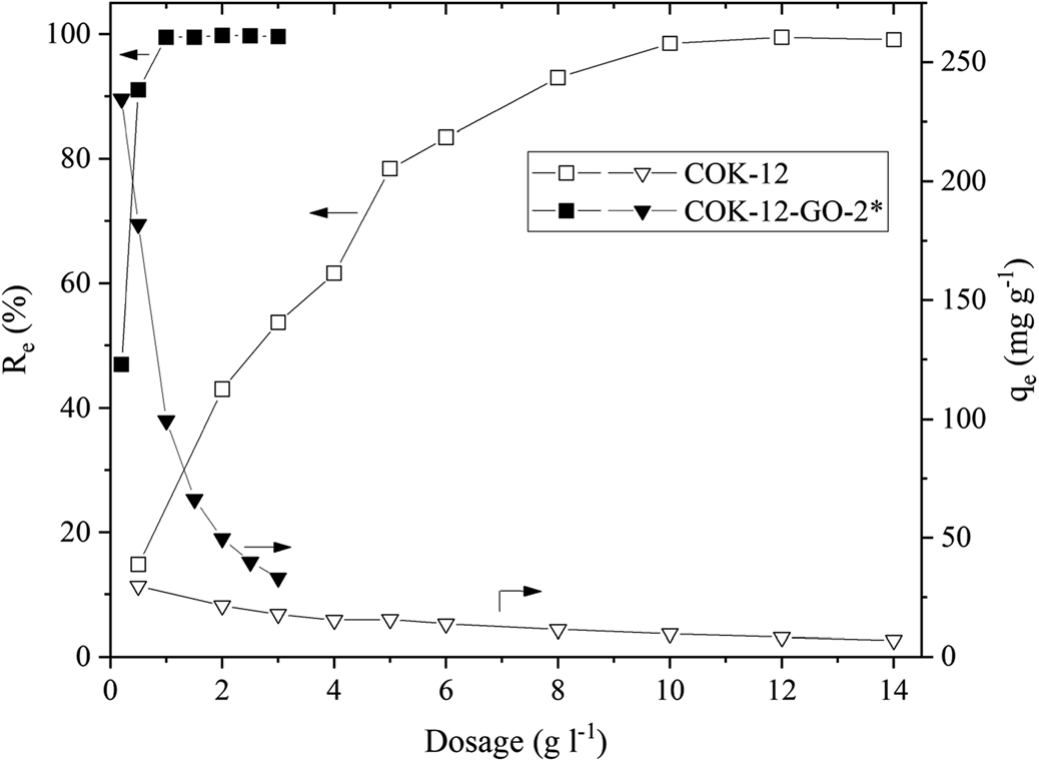
Effect of adsorbent dosage on the removal efficiency (squares) and adsorption capacity (triangles) of MB on pure COK-12 (hollow symbols) and GO grafted COK-12-GO-2* (filled symbols). Adsorption conditions were pH = 5.65, *C*_i_ = 100 mg L^−1^, *t* = 180 min.

The effect of the adsorbent dosage on the adsorption process of MB on COK-12 and COK-12-GO-2* is depicted in Fig. 5. Naturally, the equilibrium adsorption capacity decreases with increasing adsorbent dosage as not all adsorption sites can be saturated at higher adsorbent dosages. At the same time the removal efficiency increases with increasing dosage until it reaches a plateau at 100% as a higher amount of adsorbent comes along with an increased amount of active sites. This plateau is reached at dosages of 10 and 1 g L^−1^ for COK-12 and COK-12-GO-2*, respectively. Corresponding equilibrium adsorption capacities of 9.8 and 99.5 mg g^−1^ were determined at these dosages for COK-12 and COK-12-GO-2*, respectively, indicating that the grafted material is ten times more effective than the pure COK-12. With a dosage of 0.2 g L^−1^ an equilibrium adsorption capacity of 234.6 mg g^−1^ could be achieved for COK-12-GO-2*. However, adsorbent dosages of 5 and 0.5 g L^−1^ for COK-12 and COK-12-GO-2*, respectively, were chosen for further experiments because of the high removal efficiency as well as adsorption capacity at these adsorbent dosages.

#### Effect of solution pH

**Fig. 6:**
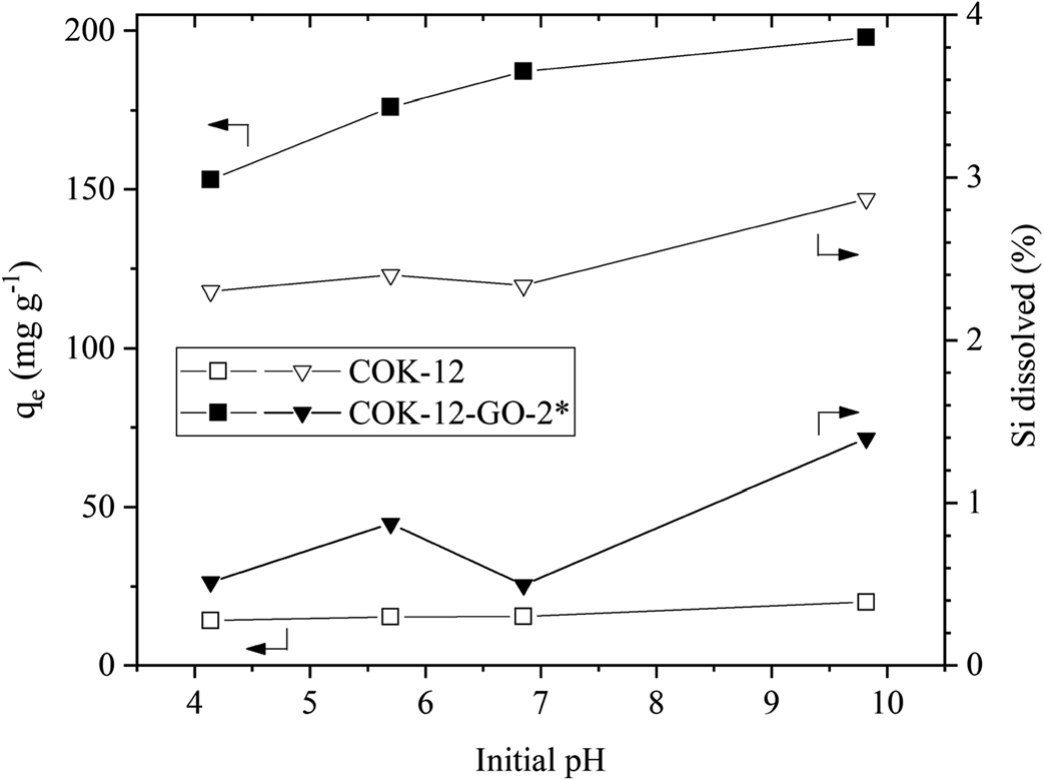
Effect of dye solution pH on the adsorption capacity (squares) of MB and stability of the materials (triangles) for pure COK-12 (hollow symbols) and GO grafted COK-12-GO-2* (filled symbols). Adsorption conditions were dosage = 5 and 0.5 g L^−1^ for COK-12 and COK-12-GO-2*, respectively, *C*_i_ = 100 mg L^−1^, and *t* = 180 min.

The effect of the solution pH on the adsorption properties of MB on COK-12 and COK-12-GO-2* is depicted in Fig. 6. It can be seen that the equilibrium adsorption capacities are stable in the range of quasi neutral pH values and slightly increase for basic pH values. As could be seen from the results from zeta potential, compare Fig. S4,† the samples possess a negative surface charge for pH values above their isoelectric point, *i.e.*, above pH 2.8 and 2.4 for COK-12 and COK-12-GO-2*, respectively, thus, electrostatic attractions between the samples and the cationic MB are favored.

As COK-12 like other OMS materials is known to dissolve in aqueous and basic conditions,^38,39^ the Si content was measured in the supernatants obtained from the dye adsorption experiment. The results are displayed in Fig. 6. While the pure COK-12 markedly dissolved with dissolution percentages of 2–3%, the Si dissolution of the grafted COK-12-GO-2* remains below 1% under acidic and neutral pH conditions and does not exceed 1.4% at basic pH 9.8. Thus, it can be concluded that grafting of GO on the surface of COK-12 not only increases the adsorption efficiency but also protects the silica from dissolution at strong basic conditions to a great extent. This is an important finding as most OMS adsorbents cannot be used in strong basic conditions due to their high dissolution affinity at these conditions.

### Adsorption isotherms

**Fig. 7:**
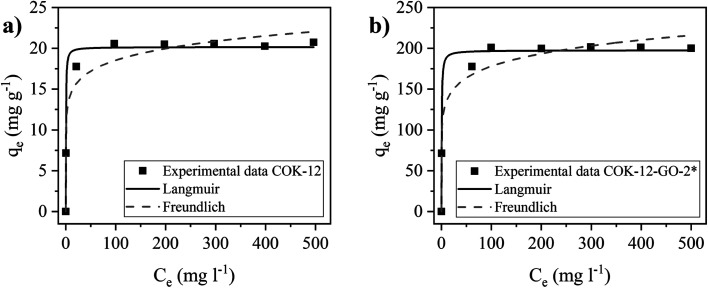
Nonlinear Langmuir and Freundlich isotherm models for the adsorption of MB on pure COK-12 (left) and GO grafted COK-12-GO-2* (right). Adsorption conditions were pH = 5.65, dosage = 5 and 0.5 g L^−1^ for COK-12 and COK-12-GO-2*, respectively, and *t* = 180 min.

The adsorption isotherms of MB on pure COK-12 and grafted COK-12-GO-2* were studied by using the nonlinear form of Langmuir and Freundlich models to fit the experimental adsorption data collected using different initial dye concentrations. Fittings of nonlinear isotherm models have been applied in order to avoid possible modification of the statistical interpretation of experimental error distributions which can result in flawed parameter variances.^40^ As shown in Fig. 7 and Table S1,† the adsorption data of both samples, COK-12 and COK-12-GO-2*, are in better accordance with the Langmuir model (*R*^2^ ≥ 0.988) compared to the Freundlich model (*R*^2^ ≥ 0.961). This suggests that the adsorption of MB on the surface of both materials is a monolayer process and not a multilayer process. This can be explained by the large size of the dye molecules, which may repel each other when they get too close. These results are in good accordance with previous works in literature which have reported that both, the adsorption of MB on GO and on silica are dominated by monolayer adsorption.^9,41^ The obtained maximum adsorption capacity of MB, *q*_m_, on the grafted COK-12-GO-2* samples is 197.5 mg g^−1^, which is much higher than that observed for the pure COK-12, 20.2 mg g^−1^, and other silica adsorbents reported in literature, Table 2. At this point it is to be noted that Liou *et al.* promote their SBA-15-GO with a maximum adsorption capacity of 242 mg g^−1^, which is not the maximum monolayer adsorption capacity obtained from the Langmuir isotherm, but a value from a kinetic experiment with unknown initial dye concentration and pH.^16^ As the maximum monolayer adsorption capacity was not initially provided by the authors, it was calculated from the given Langmuir plot to make an appropriate comparison. This calculated value of 105 mg g^−1^ shows that COK-12 grafted with GO has an outstanding maximum adsorption capacity and can be applied as an effective adsorbent for the removal of MB from aqueous solution.

**Table 2:** Comparison of the maximum monolayer adsorption capacity of MB on different silica based adsorbents considering the adsorption conditions

Material	*q*_max_ (mg g^−1^)	Adsorbent dosage (g L^−1^)	pH	Reference
COK-12	20	5	5.7	This work
COK-12-GO-2*	198	0.5	5.7	This work
SBA-15	71	0.2	7	[^42^]
SBA-15-COOH	141	10	10	[^43^]
SBA-15-GO	105^a^	0.2	—	[^16^]
MCM-41	54	1	—	[^44^]
MCM-41-TiON	129	1.6	7	[^28^]
Fe_3_O_4_/GO@mSiO_2_	125	0.2	5	[^17^]

a Calculated from given linear Langmuir isotherm plot, see Fig. 11 in ref. 16, as not explicitly provided by the authors.

### Adsorption kinetics

**Fig. 8:**
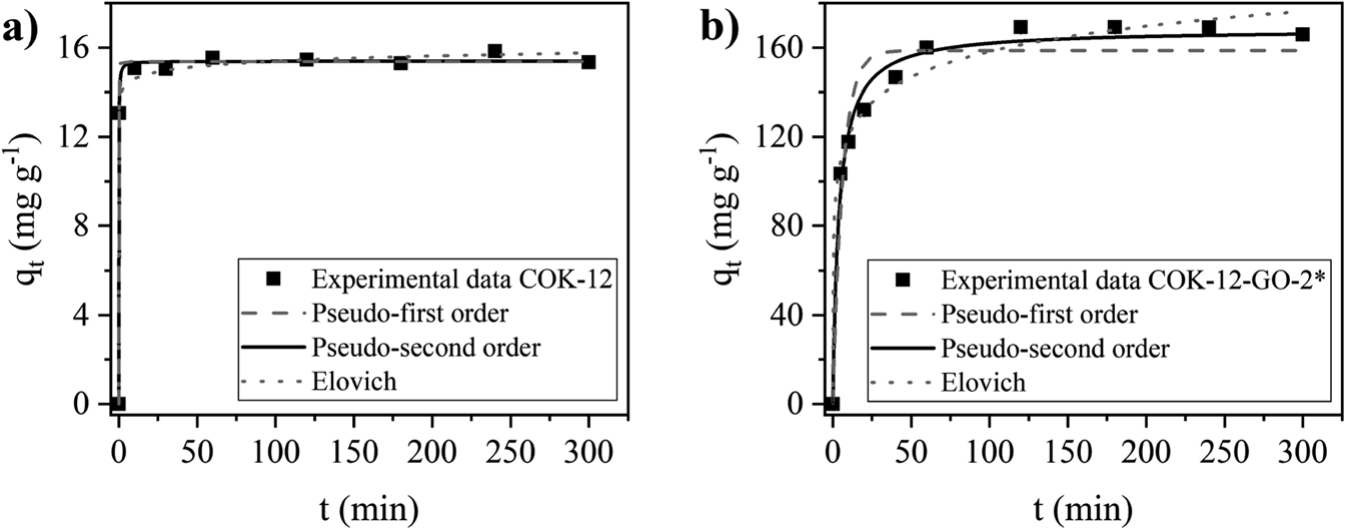
Nonlinear pseudo-first order, pseudo-second order, and Elovich kinetic fittings for the adsorption of MB on (a) pure COK-12 and (b) GO grafted COK-12-GO-2*. Adsorption conditions were pH = 5.65, dosage = 5 and 0.5 g L^−1^ for COK-12 and COK-12-GO-2*, respectively, and *C*_i_ = 100 mg L^−1^.

The kinetics of the adsorption process on MB on COK-12 and COK-12-GO-2* were studied by fitting the experimental adsorption data determined at different contact times using the nonlinear forms of the pseudo-first order, pseudo-second order, and Elovich models, as depicted in Fig. 8. The corresponding parameters of the fitting are listed in Table S2.† The experimental adsorption data of both samples reveal reasonable fittings with the pseudo-second order model as can be seen from the nonlinear regression coefficients *R*^2^ of 0.9981 and 0.9889 for pure COK-12 and GO grafted COK-12-GO-2*, respectively. Thus, it can be concluded that chemisorption is predominant as rate controlling step in the adsorption process. From Fig. 8a it can be seen that the adsorption process of MB on COK-12 is remarkably fast, as after 0.3 min, 85% of the equilibrium adsorption capacity, *i.e.*, 13.1 of 15.4 mg g^−1^, has been reached. In contrast, for COK-12-GO-2* 20 min are needed to reach a similar fraction of the equilibrium adsorption capacity, *i.e.*, 132.2 from 168.2 mg g^−1^, see Fig. 8b. The superior adsorption rate of pure COK-12 in comparison to the GO grafted COK-12-GO-2* can also be seen from the high pseudo-second order constant of 1.22 g mg^−1^ min^−1^ in comparison to the GO grafted COK-12-GO-2* with 0.00154 g mg^−1^ min^−1^. In order to ensure that the fast adsorption rate is a sample property and not an artefact of the high dosage used, the experiment was conducted with a lower dosage and a very similar COK-12 material. The results are displayed in Fig. S6 in the ESI† and confirm the exceptional fast adsorption rate of MB on COK-12. The results can be explained by the nature of the adsorption process of MB on the surface of both materials. As can be seen from Table S2,† the differences in the regression coefficients obtained from the pseudo-first order and pseudo-second order models for both adsorbents are small, which indicates that physical adsorption may also be involved in the adsorption process. Regression coefficients *R*^2^ and equilibrium adsorption capacities *q*_e_ of the aforementioned two models are similar for pure COK-12, suggesting a high physisorption contribution. Therefore, the adsorption of MB on the surface of pure COK-12 can be said to occur very fast.

### Simulated dye effluent adsorption

**Fig. 9:**
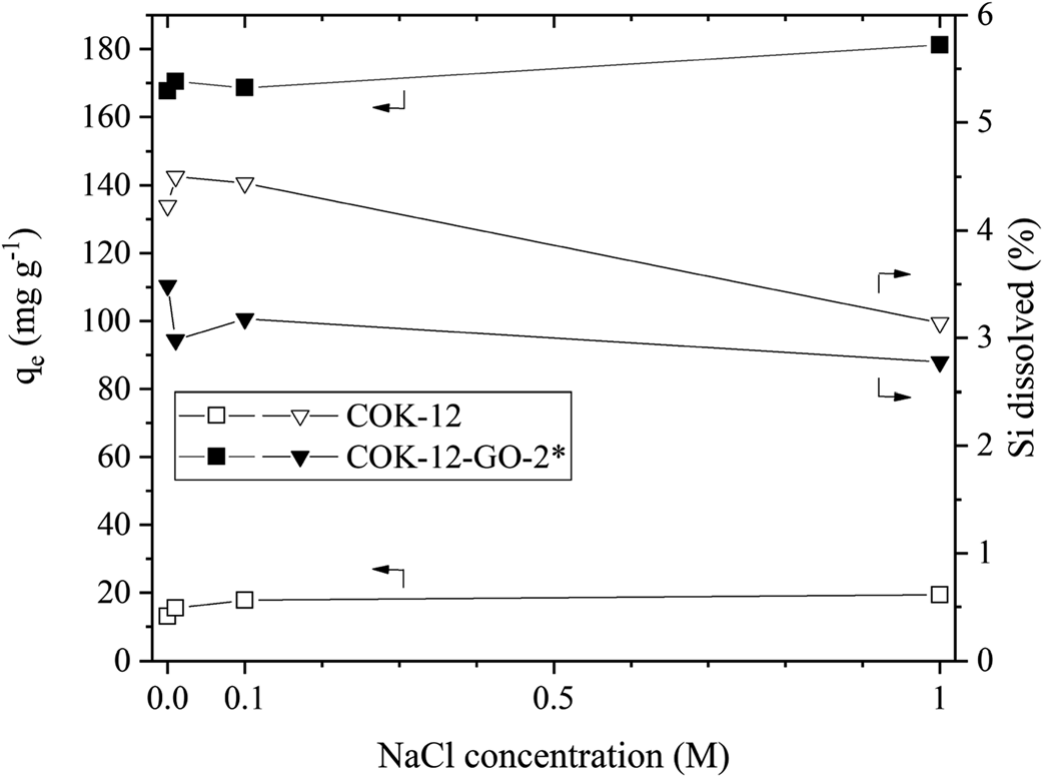
Effect of simulated dye effluent conditions on the adsorption capacity of MB on pure COK-12 and GO grafted COK-12-GO-2* (squares) and their stability (triangles). Adsorption conditions were *T* = 80 °C, pH = 5.65, dosage = 5 and 0.5 g L^−1^ for COK-12 and COK-12-GO-2*, respectively, *t* = 180 min, and *C*_i_ = 100 mg L^−1^.

The results of the simulated dye effluent adsorption are depicted in Fig. 9. From these results, two pieces of information can be obtained, the adsorption behavior at elevated temperature and the influence of the salt concentration upon the adsorption properties at elevated temperature. When comparing the removal efficiencies at 80 °C with the ones obtained at 25 °C, compare Fig. 5, it can be seen that the adsorption capacity decreases by 17 and 8% for COK-12 and COK-12-GO-2*, respectively. This phenomenon implies that the adsorption process is an exothermal reaction.^45^ The stability of both, COK-12 and COK-12-GO-2*, is decreased at 80 °C, in comparison to room temperature, an observation presented in literature for amorphous silica, however, no clear mechanism for the dissolution of amorphous silica has been published to date.^46^ Dove *et al.* published their findings about the kinetics of amorphous silica dissolution and the paradox of the silica polymorphs in which they state a model that assumes the amorphous silica presents two predominant types of surface-coordinated silica tetrahedra in solution.^47^ Furthermore, Crundwell recently developed a model based on the novel theory that proposes material removal from the surface and ion formation in solution, leaving behind charged surface vacancies.^48^ By increasing the salt concentration from 0 to 1 M NaCl, the removal efficiency (not displayed) could be increased by 33 and 7.5% for COK-12 and COK-12-GO-2*, respectively. Theoretically, the removal efficiency is expected to decrease with increasing ionic strength in systems like the one present with electrostatic forces between the adsorbent surface and adsorbate ions. Additional ions from the salt are expected to combat with the dye molecules about the existing adsorption sites of the material. However, the experimental data does not follow this convention. A number of intermolecular forces have been suggested to explain this behavior, *e.g.*, van der Waals or ion-dipole forces, which occur between the dye molecules in the solution. It is reported that these forces increase with increasing ionic strength and thus lead to higher removal efficiencies at higher salt concentrations.^49,50^ With increasing salt concentration, the stability of COK-12 remains constant and increases for COK-12-GO-2*. For amorphous silica it is reported that at room temperature the solubility of amorphous silica decreases with increasing hydration number of cations, salts like NaCl and LiCl are able to stabilize amorphous silica more efficiently than KCl for example. This is suggested to be caused by the increasing amount of free solvent that can be bound to the cation with increasing hydration number.^51^

### Dye recovery from the adsorbents

**Fig. 10:**
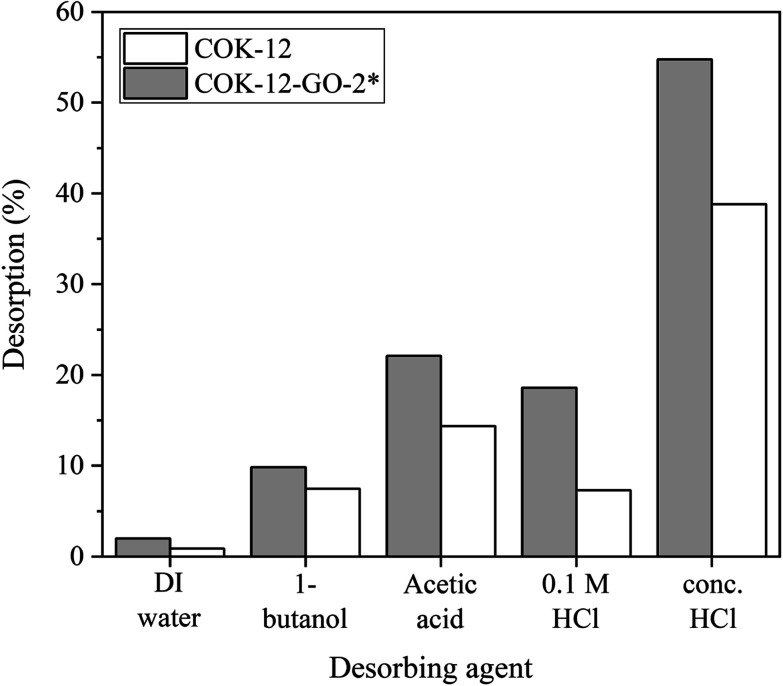
Desorption of MB from pure COK-12 and GO-grafted COK-12-GO-2*.

The results from the desorption experiments are depicted in Fig. 10. It can be seen that there is no remarkable desorption of MB from both pure COK-12 and GO grafted COK-12-GO-2* with DIW or 1-butanol. On the contrary, the usage of concentrated acetic acid and hydrochloric acid results in higher recovery efficiencies of MB from GO-grafted COK-12-GO-2* with 22 and 55%, respectively. This result can be explained by the strong interaction between the functional groups of the adsorbed MB and the functional groups on the surface of the adsorbents such as hydroxyl, carboxyl, and epoxy groups. Thus, strong acids such as concentrated acetic acid and hydrochloric acid are necessary to overcome these strong interactions in order to recover the adsorbed MB. However, the recovery efficiencies of MB from the surface of pure COK-12 adsorbents are slightly lower than those of MB from the surface of GO-grafted COK-12-GO-2* for all desorbing agents. This could be due to the possible incorporation of adsorbed MB inside the pores of COK-12, which make the recovery of the dye more difficult. In contrast, the pore volume of the GO grafted COK-12-GO-2* is lower compared to pure COK-12, wherefore MB is rather adsorbed on the surface of the GO sheets of COK-12-GO-2* than inside the pores, resulting in a better accessibility and thus, easier recovery of the dye.

### XPS study of MB adsorption

**Fig. 11:**
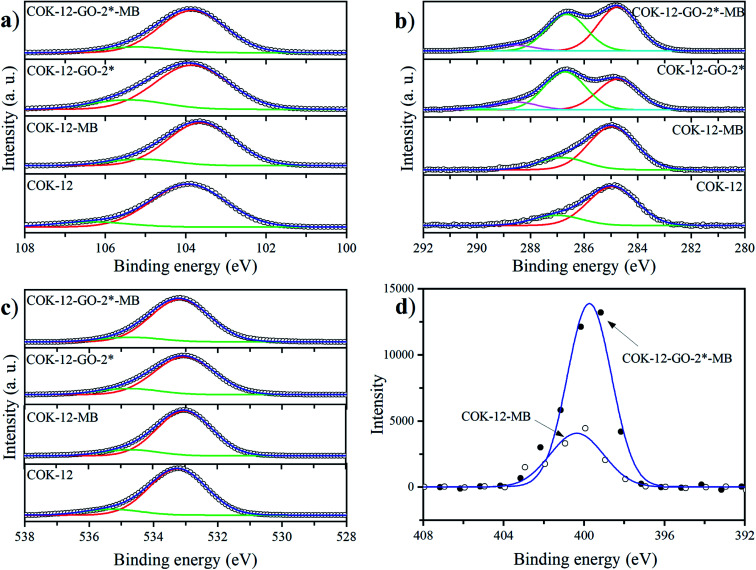
High resolution XPS spectra of (a) Si2p, (b) C1s, (c) O1s for pure COK-12, pure COK-12 after MB adsorption (-MB), GO grafted COK-12-GO-2*, and GO grafted COK-12-GO-2* after MB adsorption (-MB), and XPS spectra of (d) N1s for pure COK-12 and GO grafted COK-12-GO-2* after MB adsorption.

The MB adsorption on pure COK-12 and GO grafted COK-12-GO-2* was studied by XPS and the obtained deconvoluted Si2p, C1s, O1s and N1s spectra are depicted in Fig. 11a–d, respectively. When considering the pure COK-12, there is a little peak shift towards lower binding energies for the Si2p (103.9 to 103.6 eV) and O1s (533.2 to 533.0 eV) spectra when comparing the spectra before and after the MB adsorption. These shifts in the binding energies indicate a change in the chemical environment of Si and O on the surface of COK-12 after the adsorption of MB, which is consistent with the interaction between Si–OH/Si–O^−^ on the SiO_2_ surface and functional groups of MB. When considering the GO grafted COK-12-GO-2* before and after MB adsorption, the Si2p peak does not change its position (103.8 eV), but the O1s peak is shifted towards slightly higher binding energies (533.0 to 533.2 eV). This indicates that MB is mainly adsorbed on the surface of GO sheets, not on the COK-12 substrate. Furthermore, when considering the C1s spectra of GO grafted COK-12-GO-2* before and after MB adsorption, the intensity of the C–O peak at 286.7 eV is lower after MB adsorption. These results are in good agreement with those of the adsorption kinetics, which revealed that physical adsorption is the main process on the surface of pure COK-12, while chemisorption is the main interaction process for the GO grafted COK-12-GO-2*. The overall successful adsorption of MB on both the pure COK-12 and the GO grafted COK-12-GO-2* can be derived from the distinct peak around 400 eV in the N1s spectra.^52^ Thereby, the much higher intensity of the N1s peak from the GO grafted sample tends towards a higher uptake of MB in comparison to that of the pure COK-12. This is supported by the atomic percentages for nitrogen provided from the XPS peak table of the measurement, which amount to 0.34 and 1.12 at% for the pure COK-12 and the GO grafted COK-12-GO-2*, respectively.

## Conclusion

In summary, COK-12/GO adsorbents were prepared using an inexpensive and rapid surface grafting process, which was successfully upscaled by a factor of five. Specific surface areas between 227 to 554 m^2^ g^−1^ and a negatively charged surface over a wide pH range could be generated. Due to the higher number of functional groups provided by the GO, the adsorption capacity of the cationic dye MB on the GO grafted COK-12 is superior compared to the pure COK-12. In order to achieve removal efficiencies of 100%, 10 g L^−1^ from the pure COK-12 and only 1 g L^−1^ of the GO grafted COK-12-GO-2* were necessary at pH 5.65 and MB concentration of 100 mg L^−1^. The calculated maximum adsorption capacities were found to be 20 and 198 mg g^−1^ at dosages of 5 and 0.5 g L^−1^ for COK-12 and COK-12-GO-2*, respectively, at pH 5.65 and MB concentration of 100 mg L^−1^. Therewith, the adsorption properties of GO grafted COK-12 exceed common values for other pure and functionalized OMS materials. Adsorption performance could be well preserved at simulated dye effluent conditions. Furthermore, the GO protects the COK-12 from dissolution to a great extent up to pH 10. Desorption could successfully be performed with HCl. The overall adsorption properties were shown to be superior to that of other pure and functionalized OMS materials. In conclusion, this work showed that GO grafted COK-12 is a promising adsorbent for the cationic dye MB, combining the high specific surface area of the COK-12 substrate with a variety of oxygenated functional groups from GO while overcoming the necessity of high speed centrifugation.

## Conflicts of interest

There are no conflicts to declare.

## Supplementary Material

RA-009-C9RA05541J-s001
