# Supplementary material for: Grafting and stabilization of ordered mesoporous silica COK-12 with graphene oxide for enhanced removal of methylene blue
Source: RSC Adv. 2019 Nov 7;9(62):36271–84. doi: 10.1039/c9ra05541j (PMC9074999; doi:10.1039/c9ra05541j)
Supplement: RA-009-C9RA05541J-s001 [file RA-009-C9RA05541J-s001.pdf]

Electronic Supplementary Material (ESI) for RSC Advances.
This journal is © The Royal Society of Chemistry 2019

Supporting Information

Grafting and stabilization of ordered mesoporous silica COK-12 with graphene oxide for enhanced removal of methylene blue

Laura M. Henning[a*], Ulla Simon[a], Aleksander Gurlo[a], Glen J. Smales[b], and Maged F. Bekheet[a]

[a] Fachgebiet Keramische Werkstoffe / Chair of Advanced Ceramic Materials, Institut für Werkstoffwissenschaften und -technologien, Fakultät III, Technische Universität Berlin, Hardenbergstr. 40, 10623 Berlin, Germany

[b] Bundesanstalt für Materialforschung und -prüfung (BAM), Division 6.5 – Polymers in Life Sciences and Nanotechnology, Unter den Eichen 87, 12205 Berlin, Germany

* corresponding author. Contact details: e-mail address: laura.m.henning@ceramics.tu-berlin.de, telephone: +49 (0) 30 314 25202

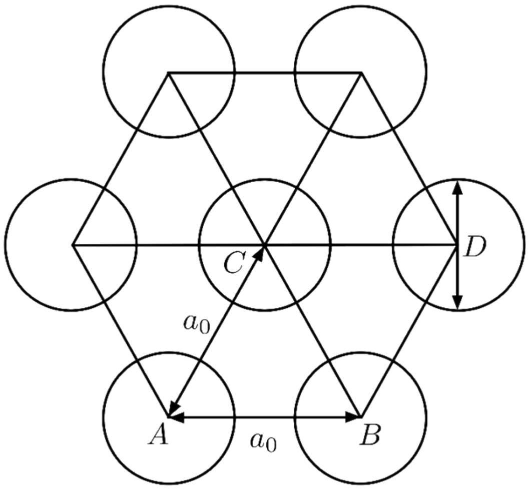

$$Area\ of\ ABC\ equilateral\ triangle = \frac{a_0^2\sqrt{3}}{4}$$

$$Wall\ area\ in\ ABC\ triangle = \frac{a_0^2\sqrt{3}}{4} - \frac{\pi D^2}{8}$$

Figure S1. Schematic drawing of the hexagonal pore structure of COK-12 and the corresponding calculation for the wall area.

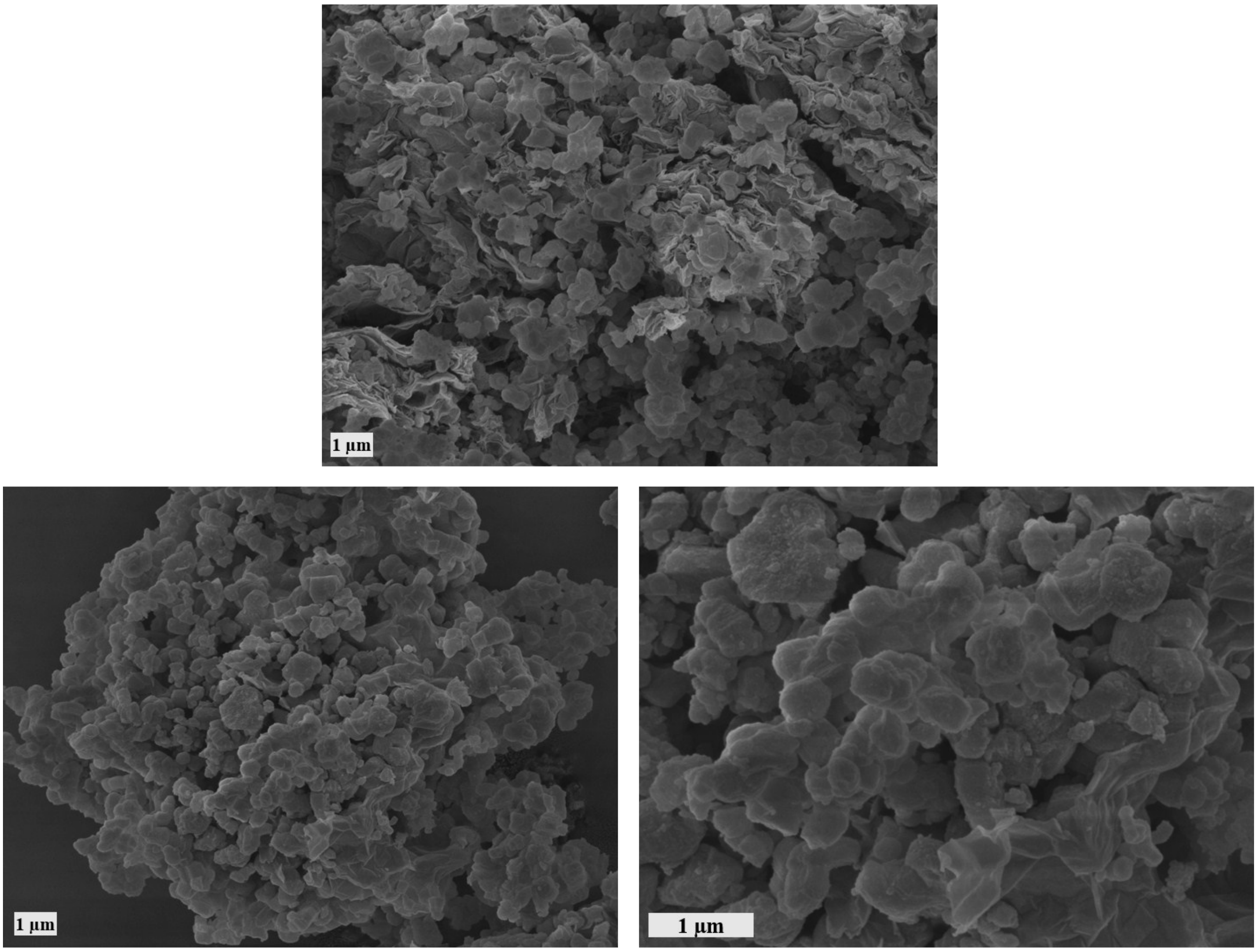

Figure S2. SEM images of GO-grafted COK-12-GO-2*.

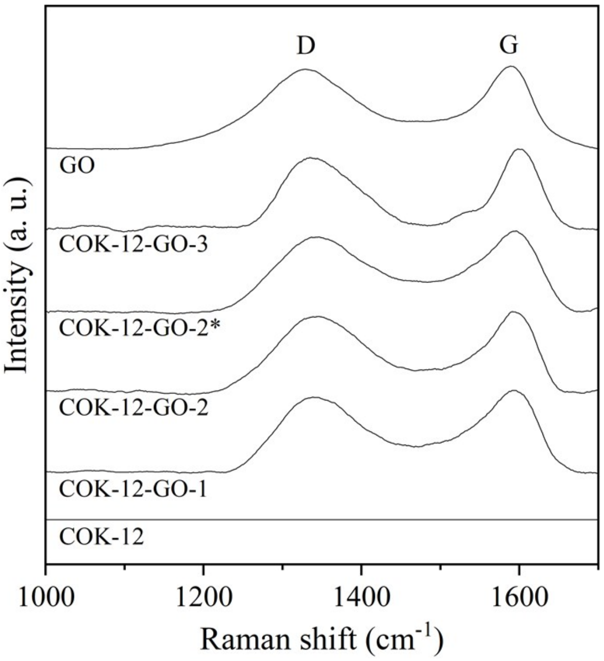

Figure S3. Raman spectra of GO, pure COK-12, and GO grafted COK-12-GO with low (-1), medium (-2) and high (-3) GO concentration. The asterisk (*) represents the upscaling.

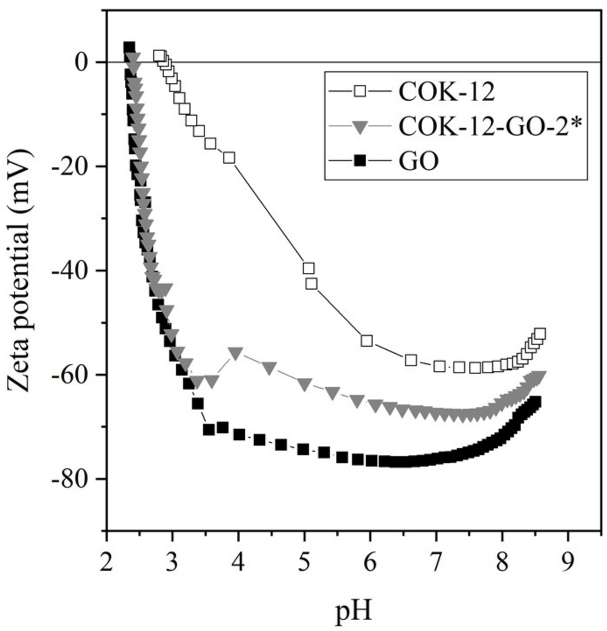

Figure S4. Zeta potentials of GO, pure COK-12, and GO grafted COK-12-GO-2*.

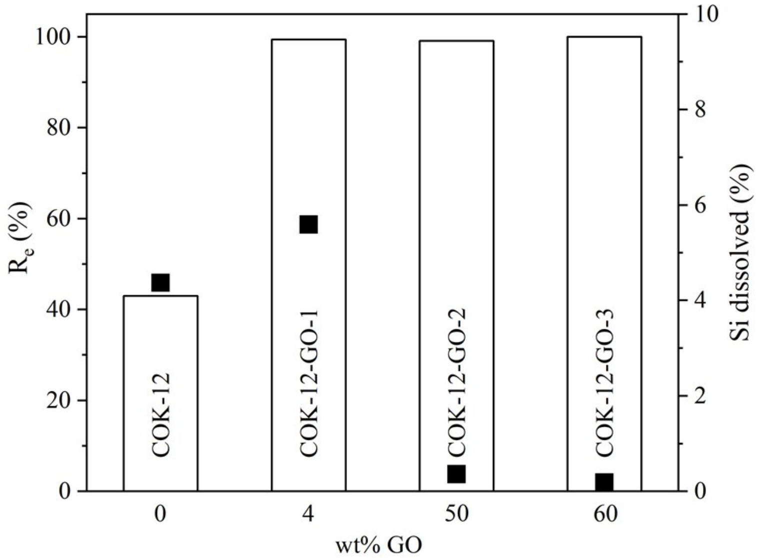

Figure S5. Removal efficiency (bar) and Si content (black square) of the supernatant after adsorption of MB on pure COK-12 and grafted COK-12-GO samples with 4 wt% (-1), 50 wt% (-2), and 60 wt% (-3) GO. Adsorption conditions were 2 g l^{-1}, 100 mg l^{-1} MB, pH 5.65, 180 min.

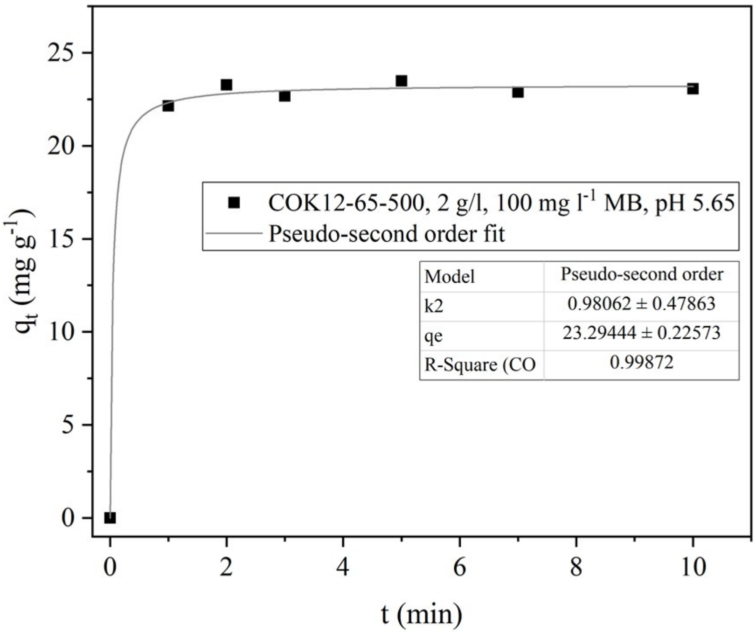

Figure S6. Nonlinear pseudo-second order kinetics fitting for the adsorption of MB on COK12-65-500, which is COK-12 but aged at 65 °C. Adsorption conditions were pH = 5.65, dosage = 2 g l^{-1}, and C_i MB = 100 mg l^{-1}.

Table S1. Parameters of the isotherm studies according to the Langmuir and Freundlich models depicted in Figure 10 for pure COK-12 and the GO grafted COK-12-GO-2*.

Model	Parameter	COK-12	COK-12-GO-2*
Langmuir	q_m (mg g^{-1})	20.2	197.5
	K_L (l mg^{-1})	2.97	2.09
	R^2	0.988	0.990
Freundlich	n (mg g^{-1})	9.30	8.35
	K_F (mg g^{-1}(l $mg^{-1})^{1/n}$)	11.30	102.52
	R^2	0.961	0.970

Table S2. Parameters of the kinetic studies according to the pseudo-first order, pseudo-second order, and Elovich model depicted in Figure 11 for pure COK-12 and GO grafted COK-12-GO-2*.

Model	Parameter	COK-12	COK-12-GO-2*
Pseudo-first order	q_e (mg g^{-1})	15.4	158.6
	K_1 (min^{-1})	6.35	0.16
	R^2	0.9976	0.9510
Pseudo-second order	q_e (mg g^{-1})	15.4	168.2
	K_2 (g mg^{-1} min^{-1})	1.22	1.54×10^{-3}
	R^2	0.9981	0.9889
Elovich	α	1.25×10^{17}	2449
	β	2.93	0.06
	R^2	0.9960	0.9855
